# The Impact of Entrepreneurial Spirituality on Business Performance: Based on the Survey of Private Enterprise Executives in Fujian China

**DOI:** 10.3389/fpsyg.2022.900852

**Published:** 2022-06-09

**Authors:** Huiling Lin, Weiqiong Fang, Guojiang Wei

**Affiliations:** ^1^Zhangzhou City College, Zhangzhou, China; ^2^School of Economics, Fujian Normal University, Fuzhou, China

**Keywords:** entrepreneurial spirituality, enterprise performance, Chinese enterprise executives, SEM, business performance

## Abstract

Entrepreneurship is the main engine of economic development. This paper aims to explore the impact of high executives’ entrepreneurial spirituality (ES) on the business performance in China under the background of “mass entrepreneurship and innovation.” By analyzing the relevant literature of entrepreneurship, the connotation and elements of ES are determined. According to the relevant research, we design the questionnaire of ES and business performance. The questionnaire was distributed to 100 private enterprise entrepreneurs by equidistant sampling, and 74 valid questionnaires were recovered. According to the questionnaire, responsibility has the highest score, followed by innovation, proactiveness, and risk-taking. The ES of women is slightly higher than that of men. The entrepreneurs with higher education also have higher score in ES. After analyzing the impact of different elements of ES on enterprise performance using structural equation modeling (SEM) and regression equation model, it is found that: (1) ES has a positive impact on enterprise performance. (2) Different factors of ES have different effects on business performance, and innovation and proactiveness play the greatest role. (3) There is an inverted U-shaped relationship between innovation, risk-taking, and business performance.

## Introduction

Entrepreneurship is the main driving force of innovation and sustainable development and one of the most key factors of economic growth (Imran et al., 2019). Under the guidance of a series of policies of “mass entrepreneurship and innovation,” China’s entrepreneurial practice has shown unprecedented active. On average, a startup company is born every 7 s, which has become the second largest venture capital market in the world (Peipei, 2021). Entrepreneurs are regarded as the spokesperson of the organization and undertake the responsibilities of clarifying objectives, identifying opportunities, integrating resources, and formulating strategies (Renko et al., 2015; Leitch and Volery, 2017). Entrepreneurs with entrepreneurial spirituality (ES) can lead enterprises to quickly adjust their organizational structure and processes, relieve environmental contradictions, and tensions under the complex situation of rapidly changing business environment (Volery et al., 2015), gain competitive advantage in the process of entrepreneurship and operation (Miller, 1983). ES is therefore seen as a powerful predictor of a successful entrepreneur (Margaa et al., 2020).

Therefore, under the background of “mass entrepreneurship and innovation,” the impact of ES on the business performance of Chinese enterprises is very worthy of discussion and research. This study will verify the impact of various dimensions of ES on business performance. The research conclusion will help to understand the impact of the important characteristics of ES on the business performance of Chinese enterprises more comprehensively.

## Theoretical Background and Hypotheses

### Performance of Enterprise

The growth of enterprise is a continuous process of using different production resources to improve efficiency (Coase, 1974). When enterprises face environmental changes, how to create value is the main factor affecting the growth of enterprises (Slater and Olson, 2000), and performance is the general goal of business activities. In enterprise management, business performance needs to be measured quantitatively in order to understand how each department works and meet the stakeholders’ needs to be informed about the business situation. By reflecting the performance of the enterprise through quantitative indicators, the operation of the enterprise can be more clearly revealed, so that managers can more accurately understand the actual situation of the enterprise and take specific measures to address the problems. Singh and Mitchell (2005) measure the growth in terms of sales, market value, and return on investment, but Tan and Mahoney (2007) measures the growth using the number of employees invested in the company.

Financial indicators can only reflect the current operating conditions of enterprises, which is the reaction of a time-point. However, the operation activities of enterprises are a dynamic development process, and the financial indicators can not reflect the deeper influencing factors and better predict the future development trend. Venkatraman and Ramanujam (1986) argue that the entrepreneurial performance of a firm is measured by a combination of financial and non-financial indicators. The non-financial ones include the quality of the product or service, the strength of the firm in developing new products, the speed of implementing policies into practical actions, etc. Covin and Slevin (1989) measure business performance *via* two aspects: one is the economic remuneration and profitability of the business process; the other is the factors in the development of the company that cannot be directly measured by financial indicators, such as the efforts of employees and the accumulation of technology, which are called growth indicators.

Combined with the above scholars’ division of business performance dimensions, this paper will measure the business performance of enterprises from two aspects: market and finance. Profitability is the key to a firm’s survival, therefore, Slater and Olson (2000) measures the financial performance of enterprises by profitability. We agree with and adopt this indicator. What best reflects a company’s growth is its market performance, which is examined in this paper from four aspects: market competitiveness, number of new customers, sales, and market share.

### Entrepreneurial Spirituality

Entrepreneurial spirituality is centered on the identification, distribution, and utilization of entrepreneurial opportunities (Covin and Slevin, 1991; Gavril, 2018), and it is the ability of entrepreneurs to explore in new ways to create social value by stimulating social change (Schumpeter, 1934), including the positioning of opportunities, creativity, the use of feedback and teamwork skills (Toma et al., 2017). ES exists not only in the entrepreneurial process, but also in companies at all stages. It is a method for entrepreneurs to think and reason (Timmons and Spinelli, 2006). Wickam et al. (2020) considered that ES is a mentality held by individuals who are eager to create and implement new and improved products, processes, and services through collaboration through the literature review and the comparative analysis of the clear skills in indeed job description. ES exists in each of our genes (Gavril, 2018). When enterprises face resource constraints, entrepreneurs will become “resourceful” (Dees, 1998), and adopt forward-looking activities in response to the reactions of competitive enterprises (Miller, 1983). So ES is a kind of strategic orientation characterized by innovation, adventure, and forward deterrence (Covin and Slevin, 1991). The connotation and characteristics of ES include integrating the innovative behavior that can occur in the whole enterprise (Oviatt and Mcdougall, 2005), awareness, and the never-ending pursuit of new opportunities to create social benefits (Dees, 1998), ability to create social value by utilizing one’s own strengths (Schumpeter, 1934), taking risks in uncertain economic environment, etc. (Oviatt and Mcdougall, 2005).

Through literature review, we believe that ES refers to the ability of entrepreneurs to bring economic and social value to enterprises with the core of discovering entrepreneurial opportunities, by setting up new enterprises to engage in business activities, or developing new products, services, and markets in established enterprises, which is characterized by innovation, adventure, initiative, and responsibility.

Miller and Friesen (1978) divided enterprises into two categories according to whether enterprise managers embody ES in the process of business activities. After comparative research, they found that enterprises with ES are often the first innovators in the market. In order to bring better user experience to consumers, they constantly launch new products or services in the market. In the long run, such enterprises will form a good brand impression in the minds of consumers, and thus increase their loyalty to the company and greatly improve the competitiveness of the enterprise’s products. Enterprises with entrepreneurial spirit are good at perceiving the business environment and are capable of formulating suitable development strategies according to the external market environment (Covin and Slevin, 1998), and are more forward-looking in the process of research and development of new products, services, and technologies (Lengnickhall, 1992), which can increase the competitiveness of enterprise and then maintain the expansion or market share showing a positive impact on the overall improvement of corporate performance. Waterman and Zhong (1982) and Brockhaus (1993), or it can improve some aspect of enterprise performance, such as financial performance (Keh and Eng, 2007), technical performance (Antoncic and Prodan, 2008), enterprise growth performance (Michael Joffe, 2017), and so on.

Based on the above scholars’ research on the impact of ES on enterprise management, this paper believes that ES has an impact on enterprise performance from three aspects. First, the innovation of ES is reflected in the enterprise’s initiative to change itself after analyzing the business environment in which it operates and vigorously develops new products or services to better meet the needs of consumers so as to obtain higher market rewards. Secondly, the risk-taking and proactiveness nature of ES are reflected in the proactive behavior of a company in the face of its competitors. It acts either as a challenger to actively challenge the opponent, or as a responder to proactively launch a counterattack when the opponent launches a challenge. Third, the responsibility of ES makes enterprises operate with integrity and law-abiding principles, and focus on profit while actively engaging in public welfare activities to return to society, thus preventing the inhibiting effect of excessive risk-taking on business growth. Therefore, this paper believes that ES has a positive and positively contributing influence on the operation and development of enterprises. Based on this, this study puts forward the following hypothesis:

**Hypothesis 1.** Entrepreneurial spirituality has a positive contribution to business performance, both in market and financial performance.

### Elements of Entrepreneurial Spirituality

From the existing studies, the measurements of ES also differ based on different research perspectives and construction methods. For example, Pawitan et al. (2017) believed that ES is measured by entrepreneurial attitude (including social value, personal attributes, and goal orientation) and entrepreneurial activities (early entrepreneurial activities and established enterprise ownership). Miller measured ES by using innovation, risk-taking, and proactive behavior (foresight) (Miller, 1983), while Covin and Slevin (1989) classified ES into innovation, risk-taking, and proactiveness spirit. Wickam et al. (2020) analyze the top three concepts of ES in the five job categories in indeed job description and their literature based synonyms are implementation (31%), collaboration (24%), and creativity (18%). Antoncic and Prodan (2008) measured from a fourfold perspective: product services, renewal capabilities, innovation processes, and risk activities. Stone (2004) added autonomy and self-confidence on the basis of Miller, Sirine and Kurniawati (2018) divided ES into five dimensions: vision, faith/hope, altruistic love, meaning/calling, and membership.

The Chinese Enterprises Survey System (Lan et al., 2019) have conducted several tracking surveys on ES and found that ES in China shows new characteristics such as increased focus on integrity, responsibility, dedication, learning and innovation, and the ability to seize opportunities and strive to develop sustainable competitive advantages.

Based on Covin and Slevin’s (1989) classification of ES, combined with the survey report of Chinese Enterprises Survey System (Lan et al., 2019) and Chinese national conditions, this study will examine four dimensions of ES in China, namely innovation, risk-taking, proactiveness, and responsibility, and study the impact of different dimensions of ES on business operation.

#### Innovation

Any form of ES means change and, no doubt, innovation (Prada, 2019). Innovation is an important way for micro and small enterprises to transform into large companies (Nasution et al., 2011), and a company manager cannot even be called an entrepreneur if he refuses to take any risks and directly imitates competitors in changing technologies and product lines (Miller, 1983). Innovativeness is the innovative business activities that top management implements in the company in pursuit of a larger market. It is the cornerstone of ES, and the other characteristics are based on innovation, and it is because of innovation that they continue to be adventurous and proactive (Covin and Slevin, 1991). Toma et al. (2017) took James Dyson as an example to conduct quantitative research and the results show the importance of innovation in cultivating entrepreneurship.

The innovation of ES includes process innovation, product or service innovation, and management innovation (Nasution et al., 2011). Enterprises with innovative spirit can quickly take advantage of market opportunities to win higher reputation and competitive advantage (Miller, 1983). Any form of innovation activity or behavior needs to be reflected through the final results, which can be tangible new physical products, intangible new services, or new patents. The measurement of these results is the performance results obtained by the enterprise through innovation activities. Shan et al. (2016) found that faster innovation speed will bring higher business performance by studying the data of 153 newly start-ups. In a changing business environment, firms can only continuously introduce new products that are more in line with consumer demand, more adaptable to technological and market environmental changes, and form product advantages over competitors, and firm performance increases as entrepreneurs’ investment in innovation activities increases (Covin, 2015).

The spirit of innovation enables entrepreneurs to break through the shackles of thinking in market competition, pay attention to innovation investment, actively innovate enterprise processes, systems, management methods, and improve the ability of enterprises to obtain and convert resources. In business competition, only when enterprises continuously invest some of their resources in the R&D of products or services, and keep their products at the forefront of market demand at all times, can enterprises continue to gain advantages in the competition of products or services, obtain competitive opportunities, and obtain market returns. Based on the above analysis, the following hypothesis is proposed:

**Hypothesis 2.** Innovation in entrepreneurial spirituality has a positive effect on business performance.

#### Risk-Taking

Risk-taking is the continuation of innovation (Covin and Slevin, 1991). It is a risk-taking activity taken by enterprises in order to seek greater competitive advantage, and only innovation without the courage to take risks is a paper talk. Therefore, “willing to take risks” is one of the most prominent characteristics of entrepreneurs (Trang, 2018). Risk-taking enables companies to better identify and seize market opportunities and to act decisively to win in the marketplace. Risk-taking by entrepreneurs is not a feverish act of risk-taking for the sake of risk-taking, but is related to an entrepreneur’s willingness to engage in high-risk projects and to take bold and prudent actions to achieve business goals (Miller et al., 1984). The types of risks undertaken by entrepreneurs include commercial risk, financial risk, and personal risk (Dess and Lumpkin, 2005).

The change of industrial technology has accelerated, and the new competitive environment has led to increased risks (Zahra and Garvis, 2000). Enterprises with risk-taking characteristics are more likely to stand out from the complex and changeable competitive environment. The process of enterprises’ input-output is actually a process of putting in risks, taking risks, and reaping rewards. The spirit of adventure largely reflects the level and tendency of entrepreneurs to take risks. Entrepreneurs with a higher risk-taking spirit are more likely to adopt high-risk innovation rather than low-risk imitation when faced with high-return innovation opportunities. They are more willing to invest in potential market returns and market opportunities in their decisions and actions, and allocate resources to what they perceive as potential opportunities, and they influence their firm’s strategic choices and formulation through a higher propensity for risk-taking, such as in the entry of uncertain markets, the adoption of new technologies, the implementation of new marketing models, etc. By studying the relationship between corporate culture, individual characteristics, and firm performance, Kitchell (1995) found that the higher the entrepreneurial risk-taking spirit, the better the innovation performance of the firm. In business operation the more risk-averse entrepreneurs are, the more likely they are to drive innovation in the industry (Cucculelli and Ermini, 2013).

However, excessive entrepreneurial risk-taking may negatively affect the growth of enterprises. Overconfident and high-risk-averse executives will actively choose high-risk and high-return projects in their investment decisions, which may easily lead to corporate overinvestment and ultimately harm the company’s interests and growth (Yu, 2014). At the same time, highly adventurous entrepreneurs tend to expect higher returns from innovative activities, thus overestimating the future benefits of the innovative project and underestimating the risks of this innovative project. A longer period of time for the innovation activity to generate profits and revenues may increase the significant threat of innovation failure to the enterprises. Geller (1980) believed that a highly adventurous and innovative top management style is appropriate in “invest/grow” situations, while a moderately conservative management style is appropriate in “earn/protect” situations, and a risk-averse, highly conservative management style is appropriate in “divest/reap” situations.

**FIGURE 1:**
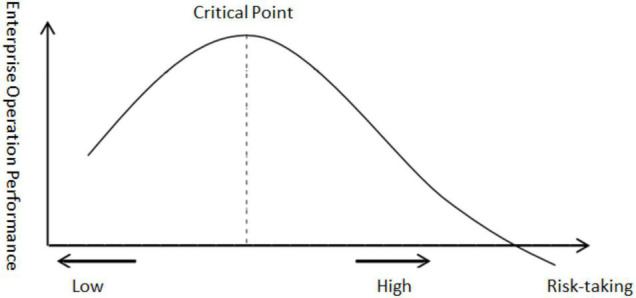
The relationship between entrepreneurial risk-taking and business performance.

This study argues that entrepreneurs need to be perceptive to seize fleeting market opportunities, so companies with a strong sense of risk-taking are more likely to be highly rewarded. However, a firm’s risk-taking capacity is not unlimited, and when the risk caused by entrepreneurs’ overconfidence is greater than its reasonable range, high corporate risk-taking will bring greater unpredictability or uncertainty to innovation projects, thus reducing business performance and acting as a disincentive to enterprises growth. As entrepreneurial risk-taking increases, business performance continues to improve, reaching a maximum at a critical point, when the critical value is exceeded, the business performance will decrease with the improvement of entrepreneurial risk-taking. The critical point varies for different specific strategic tasks (as shown in Figure 1).

Based on the above analysis, the following hypothesis are put forward:

**Hypothesis 3.** There is an inverse U relationship between risk-taking in entrepreneurial spirituality and business performance.

#### Proactiveness

Proactiveness is reflected in the manager’s initiative in the process of business activities, when competing with competitors in the market, entrepreneurs prefer to be the leader of the industry, seeking the position of market leader. Lumpkin and Dess (1996) defined proactivity as the management skill of shaping the environment to introduce new products and technologies, and optimizing new products, services and operational processes, and management methods in key business areas in order to proactively exceed rivals and competitors. The environment of enterprise development is constantly changing. When faced with business opportunities in the market, companies that react quickly to seize the opportunities and put in practical activities can have a first-mover advantage that helps improve business performance. Therefore, the characteristics of proactivity are to actively attack competitors (Covin and Slevin, 1991), so that the company can gradually influence and even control the process of adapting to the external environment, rather than being led by the nose by the external environment (Miller, 1983).

Proactiveness is an opportunity-seeking, forward-looking perspective that translates business management from the level of thought to the execution of actual activities (Nasution et al., 2011). Nasution et al. (2011) listed creativity, problem prevention, effective communication, adaptability, future orientation, implementation of new processes, and introduction of new products or services as signs of proactivity, and that companies can only achieve greater economic returns by being aggressive and pioneering. There is a significant relationship between the proactivity of ES and entrepreneurial activities. The proactivity of ES helps enterprises take advantage of market opportunities to obtain economic returns and improve their market competitive position (Kickul and Gundry, 2002).

Proactivity includes adjusting the existing competitive strategy plan, such as changing the enterprise’s competitive strategy according to the internal and external environment, or cutting off some unprofitable departments, and then planning to restructure the existing departments. On the other hand, it is a strategic orientation based on the needs of the enterprise, under the premise of fully analyzing the market situation, considering the improvement of the existing equipment and human resources management system, as well as the introduction of advanced management experience and knowledge system in conjunction with the enterprise’s own needs, and strengthening the learning of business practices and new business models, etc. Based on the perspective of business strategy, combined with strategic management theory and ES, breaking the existing technology market, seeking new knowledge, and seizing the opportunity from the strategic advantage and strategic business strategy with the exploration process of launching new products and technologies from the existing organization can effectively promote a higher level of enterprise performance. These two business strategies can give play to the positive impact of entrepreneurial resources on performance. Therefore, entrepreneurial activities should be carried out when identifying and effectively seizing market opportunities. On this basis, enterprises should actively adopt new strategies and make full use of the company’s existing resources to promote their own development.

In the process of entrepreneurial activity, the organization will face many environmental obstacles, but the entrepreneurial spirit of proactivity will help the organization to seek opportunities from the outside and find possible paths to break the bottleneck of development. Enterprises can actively adjust the internal resources of the organization to overcome the obstacles of the external environment. Therefore, we believe that proactivity can overcome the obstacles in the process of entrepreneurial development, so as to improve the level of entrepreneurial performance.

Based on the above analysis, the following hypothesis is proposed:

**Hypothesis 4.** The initiative in entrepreneurial spirituality has a positive effect on the performance of enterprises.

#### Responsibility

In the process of survival and development of enterprises, in addition to maximizing the interests of shareholders for the purpose of profit, the pro-social motivation of enterprises makes entrepreneurs connect the development of enterprises with society (Gjorevska, 2019), coordinate and meet the needs of various stakeholders, including the community and the public (Freeman, 1984). The responsibility of ES refers to the responsibilities that entrepreneurs should undertake in business and management activities, as well as the negative consequences that entrepreneurs need to bear because they fail to perform their corresponding responsibilities. Carroll proposed that corporate social responsibility consists of four aspects: economy, law, ethics, and voluntariness (Carroll, 1979). In the later research, he changed the voluntary responsibility to charitable responsibility (Carroll, 1991), and ranked the four responsibilities according to their importance, and proposed the famous “Pyramid Model.” He believed that the primary responsibility of entrepreneurs is to create profits for shareholders, so economic responsibility accounts for the largest share of these four responsibilities and is at the bottom of the pyramid, followed by legal responsibility, ethical responsibility, and philanthropic responsibility at the top. The most important function of enterprises is to provide products and services to social members, and drive social progress through their own economic development. Therefore, the economic responsibility of ES is to be responsible to its shareholders and ensure its sustainable economic interests and core competitiveness. With the continuous development of market economy, taking profit behavior as the purpose is the inevitable requirement of enterprises. Profit is the fundamental attribute of enterprises, and its ultimate goal is to maximize interests. Therefore, the economic responsibility of entrepreneurs must become the basic responsibility of other responsibilities. The United States Economic Commission classifies economic responsibility as providing products, job opportunities, and economic growth. Of course, the social requirements for corporate responsibility do not just stay at the economic level (Carroll, 1999). It also includes the awareness of legal responsibility and social responsibility. Legal responsibility is different from other responsibilities. It is an obligation that enterprises and legal persons must perform. It is mandatory and binding. It is also the minimum requirement of many moral standards for maintaining social order and world peace. The sense of social responsibility of ES is that enterprises take decisions and actions beyond their direct economic or technological interests (Davis, 1967).

The responsibility of ES to meet the interests of stakeholders sends a positive message to shareholders, employees, consumers, customers, the state, the community, and the public, and may serve as a restraint to overconfident and risk-averse entrepreneurs, which will promote a longer-term and stronger relationship with their stakeholders and bring benefits to the company. Therefore, based on the above analysis, the following hypothesis is proposed.

**Hypothesis 5.** The responsibility in entrepreneurial spirituality has a positive effect on the performance of enterprises.

## Materials and Methods

The purpose of this study was to explore the impact of ES on business performance. The object of this study is entrepreneurs and the unit of analysis in this study is the individual entrepreneurs. ES was investigated by questionnaire to obtain the data of innovation, risk-taking, proactiveness, and responsibility. To analyze the impact of ES on enterprise performance, we use structural equation modeling (SEM) and quantile regression equation model to test their relationship and influence coefficient.

### Questionnaire and Measurement

Entrepreneurial spirituality is the independent variable of this paper. Covin and Slevin (1989) designed nine questions to quantitatively analyze the innovation, risk-taking, and proactiveness of ES. The research shows that the questionnaire has good reliability and validity (Khedhaouria et al., 2020). However, due to the cultural differences in different countries and the different business environment and characteristics of enterprises, the expression of the questions has been adjusted to some extent, and two items have been added to measure the performance of entrepreneurs in innovation investment and market development. Combined with the responsibility scale of ES in the questionnaire designed by Chinese Enterprises Survey System (Lan et al., 2019), we selected three questions from three aspects: economic responsibility, legal responsibility, and social responsibility. The ES scale has 14 items totally.

We designed the questionnaire which comprised demographic backgrounds (e.g., gender, age, and educational level) and 19 questions which can be classified as innovativeness (inno), risk-taking (risk), proactiveness (proa), and responsibility (resp) and enterprise performance (enter). Business performance is the dependent variable of this paper. Based on the questionnaires of Homburg and Pflesser (2000) and Slater and Olson (2000), this study designs five items from two aspects of market performance and financial performance. The first 4 parts with 14 questions are used to test ES, and the last part with 5 questions is used to measure business performance. All questions were rated on a 7-point Likert scale (strongly disagree = 1, strongly agree = 7). Reliability testing of the scales was conducted before the questionnaires are distributed. The structure of questionnaire is as Table 1.

**TABLE 1:** The structure of the questionnaire.

		Items	Contents	Score
Individual background	Gender	1	Male, female	0, 1
	Age	1		
	Degree of education	1	Junior college and below, undergraduate, postgraduate	1–3
	Duration of enterprise	1		
Entrepreneurial spirituality	Innovation	4	4 subscales	1–7
	Risk-taking	3	3 subscales	1–7
	Proactiveness	4	4 subscales	1–7
	Responsibility	3	3 subscales	1–7
Business performance	Market performance	4	4 subscales	1–7
	Financial performance	1	1 subscales	1–7

### Sample Collection

The questionnaire was distributed to entrepreneurs in Fujian province in a targeted manner. The list of private enterprises started by entrepreneurs was collected through the *Directory of Industrial and Commercial Enterprises* in Fujian Province. Entrepreneurs from 50 manufacturing enterprises and 50 service enterprises were selected as the research objects according to the principle of equidistant sampling. A total of 100 questionnaires were distributed to entrepreneurs online through the Questionnaire Star (Software commonly used in China for questionnaire distribution) from 10 to 28 February 2022. A total of 74 valid questionnaires were collected. The questionnaire recovery rate was 74%. We use SPSS 22.0 for data collation. Table 2 shows means, standard deviations, and correlations of the study variables. Scale reliability was tested by calculating items for total correlation coefficients and Cronbach’s α for the overall scale. The Cronbach’s α of each subscales is greater than 0.7, and the Cronbach’s α of ES is 0.85, which indicates that all the variables meet the requirement of construct reliability.

**TABLE 2:** Descriptive statistics and variable reliability and correlation.

	*M*	SD	Cronbach’s α		1	2	3	4	5
1. Innovation	4.84	1.63	0.91	0.85					
2. Risk-taking	4.05	1.35	0.79		0.50**				
3. Proactiveness	4.59	1.41	0.89		0.83**	0.64**			
4. Responsibility	5.77	1.84	0.97		0.78**	0.44**	0.76**		
5. Market performance	4.73	1.54	0.95		0.81**	0.46**	0.80**	0.79**	
6. Financial performance	4.73	1.60			0.77**	0.42**	0.75**	0.75**	0.85**

M is the mean and SD is the standard deviation, N = 74, **P < 0.01.

### Data Analysis

From the overall distribution of four factors of ES, Chinese entrepreneurs have the lowest risk-taking score, with an average score of 3.98, followed by proactiveness with an average score of 4.53, while innovation and responsibility have higher scores, with 4.72 and 5.77, respectively. On the whole, it shows that Chinese enterprises are relatively conservative in operation and prefer stability strategy. In the process of operation, the enterprise emphasizes its reputation and ensures that it will not violate the law and morality. Today, innovation has become an important means for Chinese enterprises to compete. Entrepreneurs can take innovation as an important factor to win the market, and the innovation input and output are relatively high, which can be seen from the number of patents applied for by Chinese enterprises in recent years.

**FIGURE 2:**
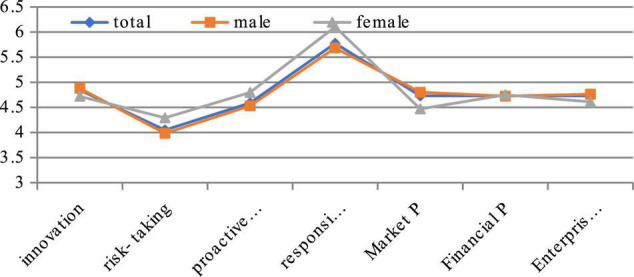
Comparison of based entrepreneurial spirituality on gender.

From the perspective of genders in the statistical data (see Figure 2). There is a certain gap between man and woman in the four aspects of ES and business performance, but it is not particularly obvious. Among the four indicators of ES, women’s innovation is slightly lower than men’s, but their risk-taking, proactiveness, and responsibility are higher than men, and the responsibility is more significant than men. However, the market performance of female executives is lower than that of male executives. On the one hand, the social capital acquisition ability of Chinese female executives is lower than that of men, resulting in low enterprise performance; on the other hand, it also shows the significance of innovation in enterprise performance in modern competition.

**FIGURE 3:**
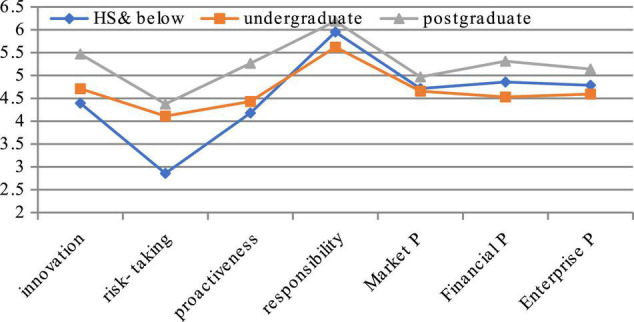
Comparison of entrepreneurial spirituality based on education.

From the perspective of the education level (see Figure 3), we can find that education of executives is important in shaping the ES. The result shows the executives with higher education get more score of ES, except certain changes in responsibility, which shows that knowledge has a stably positive effect on ES. In terms of risk-taking, there is little difference between senior executives with graduate degree or above and the executives with college students, while senior executives below senior high school have the lowest score, which shows that the operation of senior executives below senior high school is more conservative. In terms of innovation, graduate executives are much higher than others, indicating that the level of knowledge plays an important role in innovation and is an important factor to improve innovation. There is a certain gap in responsibility, but it is small.

## Hypothesis Test

This study takes the four second-order factors of innovation, risk-taking, proactiveness, and responsibility as the indicators to construct the first-order factor of ES, and the high-order factor fits well. The statistical indicators are as follows, χ^2^ = 98.20, df = 61, χ^2^/df = 1.61, CFI = 0.96, TLI = 0.95, RMSEA = 0.09, SRMR = 0.06, which shows the high-order factor can be used for subsequent analysis.

**FIGURE 4:**
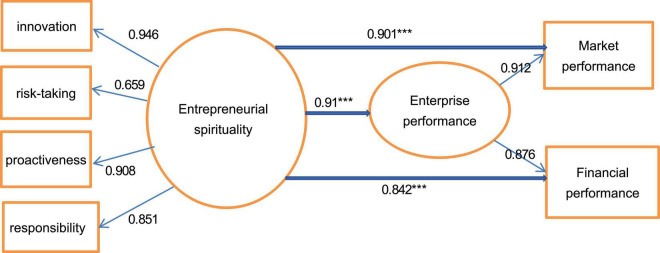
Standardized path coefficient and load factor of structural equation. ***P < 0.001.

This study used Mplus 8.3 software to test the relationship between ES and business performance by (SEM). In the measurement of Structural Equation Modeling, the fitting index is: χ^2^ = 220.74, df = 129, χ^2^/df = 1.71 < 3, CFI = 0.94, TLI = 0.93, RMSEA = 0.10, SRMR = 0.06. Except that RMSEA is slightly greater than 0.08, other indicators meet the criteria, which shows the model is suitable. On the whole, the fitting of the model is good. In the structural model, the standardized path coefficient of ES and market performance is γ = 0.901 (*P* < 0.001), and the standardized path coefficient of financial performance is γ = 0.842 (*P* < 0.001). Therefore, ES has a significant positive impact on the market performance and financial performance of enterprises. The specific loading factors and path coefficient are shown in Figure 4.

Analyzing the loading factor of ES, we can conclude innovation has become the most important factor, with the value of 0.946, followed by proactiveness, with the value of 0.908, and loading factor of the risk-taking is the lowest, with the value of only 0.659. The two loading factors of market and financial on enterprise performance are both high, which are 0.912 and 0.876, respectively. From the path coefficient, we find ES is an important variable to determine enterprise performance, and its coefficient is 0.91, which is very significant. According to the decomposition of load factors to path coefficient, the contribution of innovation to enterprise performance is 0.256 [0.91 × 0.946/(0.946 + 0.659 + 0.908 + 0.851)], the risk-taking is 0.178, the proactiveness is 0.246, and the responsibility is 0.230. The four components of ES have positive effect on enterprise performance, but the effect is different. The results fully prove that ES is an important factor determining the business performance of enterprises, which is consistent with the previous hypothesis 1, 2, 3, 4, and 5. Simultaneously, the contribution of ES to market performance is more significant than financial performance, the path coefficient is 0.901, and is significant at 1% level, and the standardized path coefficient to financial performance is 0.842, also significant at 1% level. ES performs better in market performance, because innovation and proactiveness are easier to seize market opportunities and obtain better market performance. But from the short-term analysis, market performance is not directly reflected in financial performance, so the coefficient of financial performance is lower.

In order to further verify the hypothesis proposed in this paper, we also use regression model to analyze the role of different components of ES. The regression model is as Eq. 1.


(1) $$performance=\alpha_{0}+\alpha_{1}inno+\alpha_{2}risk+\alpha_{3}proa+\alpha_{4}resp+\alpha_{5}gen+\alpha_{6}age+\alpha_{7}edu+\mu$$

In Eq. 1, *inno, risk, proa, resp, gen, age*, and *edu* is innovation, risk-taking, proactiveness, responsibility, gender, age, and education, and the last three are control variables.

Before regression, we use Stata 16.0 software to carry out multicollinearity test on the variables, and the results are shown in Table 3. It can be seen from the table that the mean value of the variance inflation factor (VIF) of all variables selected in this paper is only 3.29, which is less than the critical value 5, of which the maximum value is 4.62, which is less than the critical value 10, indicating that there is no multicollinearity problem between the relevant variables in this paper, and further regression analysis can be carried out.

**TABLE 3:** Results of variance inflation factor (VIF) of dependent variables.

Dependent variables	Innovation	Proactiveness	Risk-taking	Responsibility	Mean
VIF	3.95	4.62	1.73	2.88	3.29
1/VIF	0.25	0.22	0.58	0.35	

In the regression, we first use four independent variables for direct regression, and then add control variables for regression. The results are shown in Table 4.

**TABLE 4:** Result of the regression model.

	Market performance		Financial performance		Business performance	
C	−0.036 (−1.04)	0.317 (0.90)	−0.537 (−0.54)	0.435 (1.04)	−0.287 (−0.37)	0.376 (1.13)
Innovation	0.245** (2.11)	0.288*** (2.54)	0.283** (1.98)	0.30*** (2.22)	0.264** (2.36)	0.294*** (2.74)
Risk-taking	0.048 (0.54)	0.068 (0.75)	0.068 (0.62)	0.071 (0.66)	0.058 (0.68)	0.069 (0.82)
Proactiveness	0.433*** (3.08)	0.390*** (2.74)	0.347** (2.01)	0.348** (2.06)	0.391*** (2.88)	0.39*** (2.75)
Responsibility	0.274*** (3.10)	0.260*** (3.03)	0.285*** (2.63)	0.265*** (2.6)	0.280*** (3.29)	0.263*** (3.24)
Gender	0.439* (1.82)		0.120 (0.41)		0.279 (1.20)	
Age	0.006 (0.42)		0.023 (1.24)		0.015 (1.01)	
Education	0.178 (0.95)		0.017 (0.70)		0.097 (0.54)	
*R* ^2^	0.77	0.75	0.68	0.67	0.78	0.76
*F*	31.75	51.64	19.81	34.89	32.98	56.58

The value in brackets are t-value, ***P < 0.01; **P < 0.05; *P < 0.10.

From the perspective of market performance, in the model with and without control variables, proactiveness plays the largest role, with coefficients of 0.433 and 0.390, respectively. The role of innovation and responsibility is nearly the same, with coefficients of 0.245 and 0.274, respectively, in the model with control variables and 0.288 and 0.260, respectively, in the model without control variables. The effect of risk-taking is relatively weak, and the coefficients are 0.048 and 0.068, respectively. From the perspective of financial performance, the role of proactiveness is still the highest, and the role of innovation and responsibility has little difference. The role of risk-taking is still small and insignificant. The results of regression equation model are the same as those of SEM, but the coefficients are different, which also shows hypothesis 1, 2, 3, 4, and 5 are right.

From the perspective of control variables, gender has a certain effect on enterprise performance. Men’s effect on market performance is and financial performance is higher than women. Men are better in market than that in financial performance, with coefficients of 0.439 and 0.120, respectively. The main reason is that men’s social resources are better than women in China’s social environment, so they are more suitable to explore the market. Age has little effect on enterprise performance. Education has a higher effect on market performance, with a coefficient of 0.178, but has no significant effect on financial performance.

**FIGURE 5:**
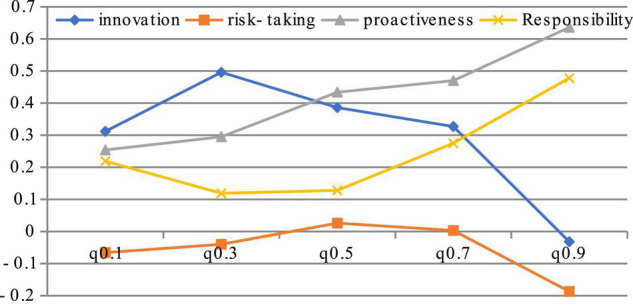
Regression coefficient of quantile regression model.

This paper believes that risk-taking can improve enterprise performance to a certain extent, but excessive risk-taking will also have a negative impact on enterprises. In order to verify the difference of the impact of risk-taking on enterprise performance, quantile regression method is used to calculate the role of four factors. The result is as Figure 5 below.

The results show that both innovation and risk-taking have the characteristics of inverted U-shape. The responsibility presents a U-shaped impact, which decreased before the 0.5 quantile, and then its positive role is becoming more and more obvious. The impact of proactiveness on enterprise performance is generally positively correlated. This empirical study further proves that hypothesis 3 is correct. The empirical results show that both innovation and risk-taking need to be controlled within a certain range in enterprise management. Although innovation is an important means of enterprise competition, the enterprises innovation must match the market capacity and financial resources of enterprise, otherwise it will also be disadvantageous. Proactiveness is very important for enterprises. It is the key to business competition to keenly discover market opportunities and take action before competitors. Therefore, the effect of proactiveness on enterprise performance is particularly significant.

## Conclusion and Implications

### Main Conclusion

(1) The innovation, risk-taking, proactiveness, and responsibility of ES have a positive role in promoting enterprise performance, which not only confirms the theory in previous literature on the promotion of ES on enterprise performance, but also confirms the theoretical view that many scholars put forward that the entrepreneurial spirit has a positive role in enterprise performance in the new era.
(2) Different elements of ES have different effects on business performance. The innovation and risk-taking of ES have an inverted U-shaped relationship with enterprise performance. The focus of enterprise management should not only emphasize innovation, but also find market opportunities in time, and one step ahead is always the key to competition. Therefore, entrepreneurs should control the risky activities in operation on the left side of the critical point in Figure 1, and the closer they are to the critical point, the more they can promote and improve the business performance of enterprises.

### Theoretical Contribution and Managerial Implications

All countries in the world need to accelerate the application of new technologies and achieve economic growth through entrepreneurship. The research on entrepreneurship focuses more on entrepreneurial resources and entrepreneurial activities themselves. This study believes that ES is an important factor to determine entrepreneurship and its performance because entrepreneurial activities are based on the will of entrepreneurs. The psychological factors of entrepreneurs have a direct impact on entrepreneurial activities. ES determines whether entrepreneurs are willing to carry out innovative business activities, which affects business performance.

To improve business performance, we need to cultivate entrepreneurial spirit, especially pay attention to innovation and proactiveness, so as to seize business opportunities in time, and accelerate the market-oriented application of science and technology. Entrepreneurs need take risks but must control the risk in certain range. The responsibility of entrepreneurs is an important manifestation of being responsible for society and enterprises. In the long run, they can be recognized by the market and improve the popularity of enterprises.

The result of the thesis shows ES is important to improve business performance. In business management, we should improve the ES, rather than simply pay attention to material resources.

#### Materialize Entrepreneurial Spirituality Into the Company’s Institutional System

Entrepreneurial spirituality is an important driving force for the operation and development of enterprises. Enterprises should pay attention to the cultivation of ES, promote managers to continuously improve their innovation awareness, pay attention to innovation investment, actively carry out business innovation and organizational innovation, take proactive actions in the face of fierce market competition environment, and actively compete for or explore the market.

#### Give Proper Play to Entrepreneurs’ Risk-Taking

The risk-taking of ES plays an important role in enterprise growth, but there is an inverted U-shaped relationship between risk-taking and enterprise performance. Therefore, we should not adopt high-risk business activities but establish a risk assessment mechanism for business activities and seek a balance between innovation and risk in business management.

#### Continuously Improve the Entrepreneurial Responsibility

Although the primary task of entrepreneurs is to pursue interests, as a member of society, enterprises must uphold the principles of integrity and compliance with the law, pay attention to the reputation of enterprises, take “prospering the enterprise, the country and the world” as their own responsibility, and actively engage in public welfare activities to repay the society. Facing resource shortage and intensified competition, enterprises should be responsible to employees and other stakeholders and protect the environment.

In short, we should effectively encourage entrepreneurs to give full play to their innovative and proactiveness of ES, constantly improve their awareness of economic responsibility, legal responsibility, and social responsibility, and give full play to their risk-taking spirit rationally, which is more conducive to promoting the development of enterprises.

### Research Limitations and Future Prospects

Due to the difficulty in distributing the questionnaire, the sample of this study is still small and the reliability and generalizability of the conclusion need to be further verified. Furthermore, this study failed to subdivide the impact of ES on enterprise performance in different industries, regions and time, and distinguish the differences and reasons.

In the future research, we can increase the sample size and conduct more in-depth analysis of the samples so as to find the differences between different regions and industries, and provide more theoretical suggestions for enterprise management.

## Data Availability Statement

The original contributions presented in the study are included in the article/supplementary material, further inquiries can be directed to the corresponding authors.

## Author Contributions

HL and GW: conceptualization, methodology, formal analysis, and writing – original draft preparation. WF and GW: software. HL, WF, and GW: writing – review and editing. All authors contributed to the article and approved the submitted version.

## Conflict of Interest

The authors declare that the research was conducted in the absence of any commercial or financial relationships that could be construed as a potential conflict of interest.

## Publisher’s Note

All claims expressed in this article are solely those of the authors and do not necessarily represent those of their affiliated organizations, or those of the publisher, the editors and the reviewers. Any product that may be evaluated in this article, or claim that may be made by its manufacturer, is not guaranteed or endorsed by the publisher.
